# How comprehensively is evidence-based practice represented in councils on chiropractic education (CCE) educational standards: a systematic audit

**DOI:** 10.1186/s12998-016-0112-0

**Published:** 2016-09-05

**Authors:** Stanley I. Innes, Charlotte Leboeuf-Yde, Bruce F. Walker

**Affiliations:** 1 School of Health Professions, Murdoch University, Murdoch, Australia; 2 Institut Franco-Européen de Chiropraxie, Ivry sur Seine, France; 3 Complexité, Innovation et Activités Motrices et Sportives, UFR STAPS, Université Paris Sud-11, Orsay Cedex, France

**Keywords:** Evidence-based practice, Accreditation, Chiropractic, Educational standards

## Abstract

**Background:**

The incorporation of evidence-based practice (EBP) is widely recognised as a necessary process for entry-level health professional training. Accreditation documents reflect the practice standards of health professions. No previous study has assessed the extent to which EBP has been taken up by chiropractic regulatory/licencing authorities, known as Councils on Chiropractic Education (CCEs), around the world. The purposes of this study were to examine CCEs’ educational standards for signs of a positive and negative approach to EBP as indicated by the prevalence and use of the words *evidence, research, subluxation* and *vitalism*, and to make recommendations if significant deficiencies were found.

**Method:**

We undertook a systematic audit of the educational standard documents of the various CCEs. CCEs were selected on the basis of the World Health Organisation. Two investigators identified the occurrences of terms explicitly related to EBP: evidence, evidence-based, research, subluxation and vitalism. This information was tabulated for comparative purposes. The date of the study was March 2016.

**Results:**

Occurrences of the term *evidence*, as it related to EBP, was highest in the CCE-Europe (*n* = 6), followed by CCE-Australia (*n* = 2), and CCE-USA (*n* = 1). None were found in the CCE-International or CCE-Canada documents. The term *research* appeared most frequently in the CCE-Europe documents (*n* = 43), followed by CCE-USA (n-32), CCE-Australia (*n* = 29), CCE-Canada (*n* = 9) and CCE-International (*n* = 8). The term *subluxation* was found only once (CCE-USA) and *vitalism* did not appear in any educational standard documents.

**Conclusions:**

Accreditation bodies are powerfully positioned to act as a driver for education providers to give greater priority to embedding EBP into entry-level programs and shaping future directions within the profession. Terminology relating explicitly to EBP appears to be lacking in the educational standard documentation of CCEs. Therefore, future revisions of accreditation standards should address lack of terminology.

## Background

Evidence-based practice (EBP) has been defined as “the integration of the best research evidence with clinical expertise and patient values and circumstances [1]” and has been widely supported and adopted across health professions. Responsible practice is generally recognised as needing to be evidence-based because it increases patient safety and makes for more efficient patient care [2–5]. EBP has been shown to reduce hospital length of stay [6], increase survival outcomes [7], improve quality of care [8], enhance data quality and its retrieval [9] and reduce financial cost [6].

Education standards have been shown to impact on the levels of evidence-based practice (EBP) in medicine and medical students’ clinical reasoning [10]. The use of EBP has been found to be directly related to the quality of the students’ classroom experience and EBP competency has been shown to be influenced by curriculum content such as training in epidemiology, biostatistics and information literacy [10, 11]. Thus maintaining high quality education and training standards with an emphasis on EBP is important for producing competent graduates. A concern is the varying uptake of EBP across health professions. Only half of United States of America (USA) medical specialities required an evidence-based basic science knowledge for accreditation while almost 80 % require proof of demonstration of the use of evidence in practice [12]. This delay in adopting EBP could potentially compromise levels of patient care. It is possible that there is similar slow uptake of EBP in chiropractic education and training with similar consequences such as lower quality of patient care.

Consideration has been given to the international implications of medical educational standards. There is evidence of varying levels of skill in medical practitioners internationally as a result of variable education accreditation standards and the concern has been raised that substandard quality may impact negatively on patient safety and levels of care with an increasingly internationally mobile workforce [13, 14]. The recent release of the World Health Organization international standards for medical education has gone someway to addressing this issue [15]. These standards ultimately intend to improve the health of all peoples. The World Health Organization global standards aim to achieve this by offering a quality template for medical education institutions and programmes for defining institutional, national and regional standards, and as a lever for reform programmes. A study comparing international chiropractic education standards looking for similarities and differences could be used as a foundation for producing a similar template for chiropractic program global standards.

### Indicators of evidence-based education standards

Educational standards adopted and monitored by educational regulatory agencies across 11 health profession bodies in Australia were compared in a recent study [16]. The authors compared the frequency of the appearance of EBP-related terms, such as the word *evidence,* in documents for informing educational programs of required accreditation standards of the need for EBP. They found that chiropractic documents used *evidence* in relation to evidence-based health care once whereas the most prolific health professions were physiotherapy (8 times), podiatry (5 times), and medicine (4 times). Pharmacy, dentistry and occupational therapy did not mention *evidence* at all. It is noted that McEvoy et al., acknowledged that the frequency of inclusion of EBP-related terms did not necessarily relate to the quality of document [16]. They also recognized that assessment of educational programs included on-site visits, interviews, reviewing of program documentation and curriculum content. However McEvoy et al, concluded that there appeared to be considerable delay in the uptake of language pertaining to EBP that would engender confidence in the adoption of EBP by professional accrediting bodies. Further, these authors state that texts relating to EBP were included infrequently and their relative absence should be rectified in future revisions of accreditation documents.

### EBP in chiropractic education

Research has shown that chiropractors are well suited to adopt and enact evidence-based practice guidelines [17, 18]. However, past research has indicated that there is scope for improvement for chiropractors in EBP [19]. Studies have identified barriers such as a lack of research skills and time to learn and adopt EBP [20, 21]. One solution proffered is the implementing of educational interventions shown to improve the use of EBP that have improved levels of patient care in other health disciplines [20]. There is evidence to suggest that adopting EBP in chiropractic education improves an intern’s perceived ability to deliver patient care [22]. Suggested remedial actions have included increased adherence to guidelines, appropriate patient advice and spinal manipulation as a first-line or adjunct treatment [19]. While there is no research evidence that regulatory change alter the standards of chiropractic practice, it would seem logical to assume that it is possible to facilitate this process by including EBP as a requirement to educational standards designed and enforced by chiropractic regulatory or licensing agencies. Regulatory and/or licencing bodies for chiropractic programs are known as Councils on Chiropractic Education (CCE). Presently there are four such bodies and each is responsible for chiropractic programs within a discrete geographical location. In addition, there is an international umbrella organisation, the International CCE (CCE-International). A question has been raised as to whether or not chiropractic educational strategies for the implementation of EBP are being adequately assessed by these agencies [23].

### Can research standards impact on EBP?

It would seem quite obvious that research is an important component of EBP and health care training standards. Nonetheless there is evidence to suggest that research is not being targeted sufficiently to inform practice and improve its quality within medicine [24]. Interventions in educational standards by regulatory authorities have been shown to have a positive impact on research in medical education if they (1) protect time for research (2) mandate mentorship and/or collaboration, (3) ensure departmental and institutional commitment and leadership, and (4) are provided with adequate financial support [25]. Consequently accreditation standards should address research, at least across these four areas, for promoting and scaffolding EBP. An audit of CCE standards with respect to the word *research* may provide insight into ways in which EBP is dealt with presently.

### The terms “subluxation” and “vitalism”

The word “subluxation” in the chiropractic context has been variously interpreted and defined. One interpretation is that subluxation of the spinal column and other articulations can affect the nervous system function and the expression of health, which may result in symptoms, infirmity and disease [26]. Others use the term to describe a musculoskeletal-based “manipulable lesion” [27]. More recently a large number of chiropractic programs in Europe, South Africa, Australia and Canada became signatories to a position statement on the vertebral subluxation complex as a vitalistic construct [28]. These programs stated that claims that it is the cause of disease is unsupported by evidence. Further, its inclusion in a modern chiropractic curriculum in anything other than an historical context is therefore inappropriate and unnecessary. Other chiropractic educators have stated that use of this term has contributed to a breakdown in communication between chiropractors and other health professionals [29].

This vitalist/subluxation construct is known to often underpin unsubstantiated claims in patient brochures [30], wellness practice based on “vitalism” tenets [31] and anti-immunization views [32]. Based on this position it can be argued that the presence of words such as “subluxation” and “vitalism” in accreditation documentation could be considered a “red flag” for inappropriate standards and be contrary to evidence-based practice.

### Comparing chiropractic educational standards internationally

Variable international practitioner profiles have been seen in chiropractic populations [32, 33]. A group of practitioners in Canada were shown to have anti-vaccination beliefs, higher levels of X-ray usage, to be less likely to receive or make referral to or receive referrals from general practitioners, and to utilize specific treatment types, some of which were considered unsuitable [33]. This practitioner profile was found to be related to a cluster of chiropractors who had graduated from certain chiropractic institutions located in the United States of America (USA). Competency standards for graduate-entry level chiropractors devised and enforced by CCEs are not the same everywhere internationally [34]. One possible explanation for these unsuitable profiles is different international standards. To avoid the acceptance of unsuitable practice profiles, as a minimum, one could expect that the CCEs would relate to topics such as evidence and research.

It is believed that a high quality medical education and accreditation system would improve the quality of medical care [35]. Further, generalized quality standards internationally may improve educational program structures and ultimately result in improved standards of care for patients worldwide as well as improved workforce mobility [36]. Studies have been undertaken for medicine with the intent of producing a single relevant robust high quality curricular resource [13–15]. No such study has been undertaken for chiropractic education.

### Aim

The aim of this systematic audit was to investigate similarities and differences between the standards provided by various CCEs in their use of words that relate to EBP, both positively and negatively. A broader examination of other key terms in educational standards will be the subject of future research.

### Objectives

The objectives were to examine CCEs’ educational standards for signs of a positive and negative approach, respectively to EBP as indicated by the prevalence and use of the words

1. Evidence
2. Research
3. Subluxation
4. Vitalism, and
5. to make recommendations if significant deficiencies were found.

## Method

A systematic audit of current educational standards was undertaken for the CCEs who are currently recognized by the World Health Organization [37]. This included the CCE-Australia, CCE-Canada, CCE-Europe, CCE-USA and CCE-International. The educational standards were downloaded from their respective websites as PDFs at the time of the study (March, 2016). This study was an analysis of website content and did not involve collecting data from human participants, hence ethics approval was not required.

The systematic audit consisted of three phases:

- searching for the words *evidence*, *research*, *subluxation, and vitalism* in the respective educational standards
- determining if the term was related to education/training
- tabulating the results for each CCE to establish any similarities and differences.

The first phase of the audit aimed to locate the specified words of interest. The PDF texts were searched using the full reader search in Adobe Reader (XI). All occurrences of the word were copied verbatim and extracted into a spreadsheet by two of the authors (SI and CL-Y). Disagreements were resolved by discussion; a third author was available if a word categorization could not be agreed upon.

The second phase of the audit aimed to determine the frequency of the use of the word in a manner that indicated if it was being directed toward training or educational standards. For example the term *evidence* was searched for to ensure that variations of the full phrase, such as EBP or EB health care, were not missed. The level of the use was then determined by following the categorization of “heading”, “text” or “other”. A “heading” was determined to be a first heading in larger font or bold indicating a section of information. “Text” was allocated when the word was found within a sentence describing a standard. “Other” was used when the word was related to an irrelevant context eg, “a program should provide evidence of financial records for the past 3 years”. Thereafter the frequency of use was established for each word and category.

In the third phase of the audit, the extracted spreadsheet was limited to the “text” words. Common themes in the sentences which the word appeared were identified. Lists were created under identified common themes. These were then compared across all CCEs for similarities and differences.

This process was repeated for the words *research, subluxation* and *vitalism*.

## Results

There were no disagreements on word categorization between the two authors during the extraction process.

### Objective One: the word *“evidence”*

Across the five CCEs, the word *evidence* appeared 85 times with nine of these being specific to EBP or health care (Table 1). This ranged from zero to six times in individual CCE documents. The term *evidence* did not appear in any major headings. *Evidence-based* appeared most frequently in the CCE-Europe educational standards (*n* = 6) followed by CCE-Australia (*n* = 2) and CCE-USA (*n* = 1). CCE-Canada and CCE-International made no mention of *evidence-based* practice or *evidence-based* health care.

**Table 1:** Frequency of words “evidence” “research” “subluxation” in CCE Educational Standards

Word		Evidence			Research			Subluxation		
	Total document word count	Heading	Text	Other	Heading	Text	Other	Heading	Text	Other
CCE-Australia	6932	-	2	14	7	29	1	-	-	-
CCE-Canada	15,200	-	-	40	6	9	-	-	-	-
CCE-Europe	16,610	-	6	20	5	43	-	-	-	-
CCE-Int	2724	-	-	5	1	8	1	-	-	-
CCE-USA	12,445	-	1	56	4	32	1	-	1	-

The CCE-Europe expected programs to teach EBP and the best use information technology in this process. The standards for the CCE-Europe stated that evidence should inform knowledge and principles of practice and keep the curriculum up to date. Documents from the CCE-Australia standards were found to have the requirement that information technology should be part of the education for “*Evidence-based health care”*. The second occurrence in the CCE-Australia standards was for the principles of “*Evidence-based health care”* to be taught throughout the curriculum. The CCE-USA expected students to be introduced to the value of EB scientific thinking.

### Objective Two: the word *“research”*

Across the five CCEs, the word *research* appeared 147 times (Table 1). Of these, 121 were embedded within the educational standard documents as prescriptive text defining research with chiropractic programs. This ranged from eight to forty three times in individual documents. Research appeared most frequently in the CCE-Europe educational standards (*n* = 43) followed by CCE-USA (*n* = 32) CCE-Australia (*n* = 29), CCE-Canada (*n* = 9) and finally CCE-International (*n* = 8). The term *research* was classified as “other” three times.

All CCEs stated that programs should train students to be good consumers of research by teaching them how to acquire, appraise and apply evidence within an environment that encourages research”. Further it was commonplace for CCE educational standards to state that students should be trained in research methods and be given the opportunity to become producers of research by conducting research projects. All CCEs expected adherence to high standards of research conduct and ethics.

However, differences emerged over the scope of research (Table 2). Two CCEs adopted the position that research should be focused on the field of chiropractic. Others did not limit it to any particular area or discipline. In contrast two CCEs specified the need for it to be conducted with other health sectors. Only one of the CCEs’ educational standards expected programs to provide opportunities for students to progress to post graduate research. Two of the five CCEs required research to inform chiropractic practice and three required it to inform education and teaching. Finally, only two of the five CCEs expected programs to provide research active staff to supervise and teach students.

**Table 2:** Comparison among CCEs of use of the term *research*

Research statement	CCE-Aust	CCE-Can	CCE-Euro	CCE-Int	CCE-USA
Must teach research inquiry & scientific method	X	X	X	X	X
Processes/policy that recognises, develops, supports staff research	X		X		X
Establish research programs	X	X	X	X	X
Provide adequate time/space/finances/resources	X		X		X
Create an environment which encourages/facilitates research	X	X	X		X
Adhere to ethics/highest standards of research conduct	X	X	X	X	X
Written policies protecting human/animal subjects	X	X		X	X
Staff development to include research	X		X		X
Research must inform chiropractic practice			X		X
Research must inform/interaction with education/teaching	X		X		X
Evidence its contribution to body of research	X	X			X
Establish objectives for research		X			X
Students to conduct research projects	X		X	X	X
Students trained in research methods	X	X	X	X	
Students to be encourage & prepare for engagement in research	X		X		x
Students have post grad research opportunities	X				
Encourage students to gain research experience	X		X		X
Compile evidence of contribution to profession	X	X		X	x
Provide research active staff who supervise students			X		X
Research with other health sectors			X	X	
Must research the field of chiropractic		X		X	
Be meaningful research & highest possible quality					X

“X” denotes the presence of the word research in the CCE educational standards in the specified context

### Objectives three and four: the words “subluxation” and “vitalism”

The word “Subluxation” was found only once (CCE-USA standards). It stated that chiropractic education programs should train its graduates to *“assess and document a patient’s health status....including subluxation/neuro-biomechanical dysfunction (pg 11 Educational Standards USA)”.*

“Vitalism” was not found in any of the CCE documents.

## Discussion

This study was the first to systematically audit all CCE Education Standards for indicators of EBP and/or EB health care using keywords that were indicators of the adoption of EBP and others that indicated a non-adoption. Although we found only some references to the key words indicative of the presence of EBP it was encouraging that the keyword “subluxation”, which we considered an indicator of an inconsistent or incomplete adoption of EBP was found only once and that “vitalism”, in our opinion, also an indicator of non-adoption of EBP was absent in these standards.

Of the five chiropractic regulatory bodies only the CCE-Europe and CCE-Australia contained statements requiring an EB approach to be taught throughout the curriculum. The CCE-USA’s only mention was the expectation that students should be introduced to scientific thinking. The support for EBP with the use of explicit terms was generally disappointing in the CCE educational standards. These documents have the potential to act as drivers for education providers to embed EBP into practice [16]. EBP has been shown to influence educational, entry-level graduates and practitioner levels of care which in turn has improved patient outcomes [38]. By extension there can be little justification for anything other than active support for the inclusion of explicit language detailing an EB approach.

Nevertheless, the word research was more widely included in CCE educational standards. There appears to be uniform agreement that research is a core component to CCE educational standards. This as reflected in the common statement that research should be established, resourced, and taught in programs. This reflects the view that programs should be, at a minimum, producing skilled clinicians who are competent consumers of research through teaching basic skills of EB medicine [39]. This “consumer of research” level of training, an ability to evaluate evidence, has been shown to be an important element of clinical competence [40]. However, some view research as a separate educational component or subject, like anatomy or physical diagnosis. Others perceive research as something which is foundational to the entire program. For example the CCE-Europe and CCE-USA see research as informing chiropractic practice, education and teaching. Interestingly, two of the CCEs (Europe and International) required research to be conducted with other health sectors, indicating a desire for collaboration, and thus integration, with the broader health care community.

Medical educators believe that evidence-based education is not possible without adequate research support and funding [41]. Likewise the present authors, and others, believe that if chiropractic education and the profession is to continue to establish itself as a credible and mature health profession then there needs to be an emphasis on not just training skilled consumers of research but also on facilitating increased numbers of the producers of research ie, the number of post graduate researchers and research active academics within chiropractic programs [42, 43]. This attitude or path is not reflected in all CCE documents. A single CCE required post graduate opportunities to be provided for students and only two CCEs stated that research active staff should be provided to supervise research students. These two provisions are examples of changes that could be implemented in all CCE educational standards, if monitored and enforced, to facilitate the increased adoption of research to scaffold EBP.

As previously mentioned the leaders of a large number of chiropractic educational programs have signed the “Clinical and Professional Chiropractic Education: a Position Statement” [28]. They describe *subluxation* and *vitalism* as being unsupported by evidence. *Subluxation* was found once whereas *vitalism* was not found in this audit. The CCE-USA adopted the position that chiropractic programs should train their graduates to be able to detect *subluxations*. This raises several questions. Should a non-evidence based construct like subluxation be mandated to be taught in an educational program via educational standards? Some chiropractic educators have made a decision not to teach *subluxation/vitalism,* other than as a historical concept, because of a lack of evidence. Should educators be supported in this evidence-based approach by regulatory/licencing agencies actively promoting the non-teaching of subluxation/vitalism by making specific mention of its appropriate context in CCE educational standards? The absence of the words *subluxation* and *vitalism* may be interpreted as either a reluctance of regulatory bodies to actively prescribe against the use of this non-evidence based agenda from chiropractic programs or that these terms are out dated, historical and of no current importance. We contend that silence on these matters is inappropriate and could open the door to chiropractic practice programs teaching a doctrinal dogma unsupported by evidence.

### Strengths and limitations

This was a comprehensive audit of the available educational standards for the CCEs recognized by the WHO. All public domain material was scrutinized. The screening method was automatic and the authors remain confident that they have found the terms and appropriately classified them according to “heading, text or other”. The search for other terms, however, could perhaps have resulted in other findings and conclusions.

It should also be borne in mind that the frequency of terms does not necessarily relate to the quality of the document. We did not include competency standards as this was studied in a previous paper [34]. Also we recognize that program evaluation extends beyond these documents alone and requires an extensive self-evaluation, inspection and review process. However, the contents of these standards are clearly the foundation for such evaluations, and are therefore important documents to scrutinize.

## Conclusion

Educational standards have the potential to act as initiators and promoters of quality educational programs by improving the competency of entry-level graduates. Relevant terms used in these documents have the potential to clearly specify what standards educators should and can teach and use for training. CCE choice of specific vocabulary in relation to evidence-based practice in their accreditation documents has been slow and incomplete in the uptake of contemporary evidence-based trends. The absence of malevolent terms such as “subluxation” and “vitalism” and firm statements about their undesirability may provide opportunities for aberrant chiropractic programs to be accredited. Future revisions of accreditation standards should address this.

## Abbreviations

CCE, council on chiropractic education; EBP, evidence-based practice; USA, United States of America
